# Supplementary material for: A Likelihood Approach for Real-Time Calibration of Stochastic Compartmental Epidemic Models
Source: PLoS Comput Biol. 2017 Jan 17;13(1):e1005257. doi: 10.1371/journal.pcbi.1005257 (PMC5240920; doi:10.1371/journal.pcbi.1005257)
Supplement: S1 File — (TAR.GZ) [file pcbi.1005257.s014.tar.gz › HSPH_Online-SI-Revision/MSS10/n10-mild/n10-mild_NEW_table8.pdf]

1	R0	{1.25046, 6.80278, 0.1}	{1.22497, 3.09134, 0.05}	{{1.22544, 3.07157, 0.}, {1.22544, 3.07157, 0.}}	{2.0388, 54.5577, 140.}	inf	102.	45.3614	{17.6031, 69.7676}	55.6584	0.415889	newly	103.	94.74	{30., 155.}	37.5922	0.211783	Reff	0.913086	0.901409	{0.897194, 0.905156}	1.2944	ar	3516.	2962.08	{2684., 3240.}	15.8572
2	R0	{1.32013, 4.45847, 0.1}	{1.30567, 4.1611, 1.32}	{{1.28596, 3.98513, 0.}, {1.31748, 4.25564, 2.}}	{1.09541, 6.66979, 1288.}	inf	44.	41.0703	{23.3623, 57.7094}	23.9671	0.117926	newly	57.	55.73	{31., 84.}	27.6316	0.130575	Reff	0.755774	0.747877	{0.73566, 0.756333}	1.08094	ar	4420.	4389.29	{4302., 4462.}	1.24638
3	R0	{1.31785, 5.36796, 1.}	{1.37622, 3.76902, 2.9}	{{1.36035, 3.22945, 1.}, {1.40116, 4.94504, 4.}}	{4.42916, 29.9255, 196.}	inf	84.	49.5289	{23.5534, 75.9929}	41.9644	0.281846	newly	97.	70.93	{35., 101.}	31.1649	0.189402	Reff	0.69253	0.727936	{0.717561, 0.74223}	5.11253	ar	4942.	4837.3	{4726., 4950.}	2.40834
4	R0	{1.38801, 6.16031, 0.1}	{1.36146, 4.93357, 1.05}	{{1.3618, 4.98002, 1.}, {1.3618, 4.98002, 1.}}	{1.91254, 19.9136, 950.}	inf	76.	45.094	{30.4538, 59.4333}	40.6658	0.240701	newly	81.	53.56	{35., 72.}	34.9383	0.200418	Reff	0.752718	0.74253	{0.740436, 0.744708}	1.35349	ar	4790.	4665.09	{4597., 4748.}	2.65073
5	R0	{1.27955, 11.57, 1.}	{1.17101, 5.77503, 1.01}	{{1.17082, 5.77111, 1.}, {1.17082, 5.77111, 1.}}	{8.48237, 50.0862, 1.}	inf	144.	63.1773	{47.8093, 77.5124}	56.1269	0.365773	newly	92.	73.02	{55., 88.}	22.	0.114752	Reff	0.976553	0.903182	{0.901358, 0.904835}	7.51324	ar	3460.	2736.84	{2510., 3007.}	20.9006
6	R0	{1.22184, 4.34395, 1.}	{1.23953, 3.00099, 0.}	{{1.22544, 2.5364, 0.}, {1.25474, 3.26445, 0.}}	{1.44748, 30.9155, 100.}	inf	60.	47.1062	{18.8989, 73.7285}	32.4004	0.204556	newly	80.	91.96	{37., 145.}	42.425	0.188279	Reff	0.820343	0.833812	{0.822556, 0.843846}	1.64837	ar	3524.	3489.15	{3332., 3691.}	3.51674
7	R0	{1.23856, 4.59068, 0.1}	{1.25773, 3.38432, 0.05}	{{1.22544, 3.07157, 0.}, {1.28596, 3.98513, 0.}}	{1.86798, 26.2785, 144.}	inf	59.	29.5248	{12.6999, 49.552}	50.4613	0.361784	newly	87.	51.46	{22., 85.}	44.7586	0.312546	Reff	0.795403	0.811422	{0.790628, 0.829942}	2.23084	ar	3855.	3670.01	{3557., 3814.}	4.90713
8	R0	{1.34531, 10.6689, 1.}	{1.26705, 3.54144, 0.}	{{1.25474, 3.26445, 0.}, {1.28596, 3.98513, 0.}}	{5.81735, 66.806, 100.}	inf	248.	59.055	{28.0219, 84.4453}	76.1875	0.664949	newly	164.	98.55	{43., 149.}	40.7378	0.273584	Reff	0.83611	0.811405	{0.800437, 0.825929}	2.9548	ar	4852.	3834.54	{3670., 4001.}	20.9699
9	R0	{1.31775, 5.19226, 0.1}	{1.34371, 4.60476, 2.03}	{{1.31748, 4.25564, 1.}, {1.3618, 4.98002, 5.}}	{1.98662, 11.3149, 1930.}	inf	73.	44.1627	{22.1561, 64.2231}	40.3032	0.253207	newly	85.	54.46	{29., 78.}	37.5294	0.234451	Reff	0.713693	0.731611	{0.717344, 0.741755}	2.51056	ar	4708.	4669.99	{4579., 4777.}	1.64635
10	R0	{1.25916, 6.89207, 0.1}	{1.23496, 3.34904, 0.33}	{{1.21159, 3.07157, 0.}, {1.25474, 3.57392, 1.}}	{2.13495, 51.4074, 364.}	inf	69.	29.9721	{10.9289, 58.3323}	56.7374	0.434714	newly	65.	53.15	{22., 101.}	44.3538	0.248136	Reff	0.845904	0.834461	{0.818164, 0.850196}	1.8037	ar	3823.	3372.93	{3254., 3502.}	11.7727
11	R0	{1.20377, 5.46566, 0.1}	{1.25417, 2.09351, 1.23}	{{1.24751, 1.35135, 0.}, {1.2663, 2.5364, 3.}}	{4.18652, 61.6971, 1248.}	inf	56.	35.4569	{7.18019, 71.5871}	48.4681	0.378418	newly	56.	108.02	{18., 194.}	125.964	0.379289	Reff	0.847334	0.885381	{0.875496, 0.896493}	4.49018	ar	3114.	3169.31	{2931., 3454.}	5.41073
12	R0	{1.27878, 2.55804, 0.1}	{1.24751, 2.5364, 0.}	{{1.24751, 2.5364, 0.}, {1.24751, 2.5364, 0.}}	{2.44507, 0.845784, 100.}	inf	4.	11.2445	{3.16149, 20.2828}	190.035	0.410206	newly	9.	23.26	{6., 45.}	172.	0.381211	Reff	0.778393	0.758457	{0.757261, 0.759371}	2.56117	ar	3930.	3957.68	{3917., 4023.}	0.865649
13	R0	{1.41227, 5.45696, 0.1}	{1.3618, 4.98002, 1.}	{{1.3618, 4.98002, 1.}, {1.3618, 4.98002, 1.}}	{3.57345, 8.74009, 900.}	inf	53.	45.4715	{27.7671, 61.9271}	22.3634	0.118496	newly	51.	53.	{32., 72.}	23.6863	0.107036	Reff	0.744831	0.71924	{0.716982, 0.721541}	3.4358	ar	4822.	4830.26	{4745., 4910.}	0.983409
14	R0	{1.32917, 7.27593, 1.}	{1.34436, 6.25107, 2.}	{{1.34436, 6.25107, 2.}, {1.34436, 6.25107, 2.}}	{1.14268, 14.0857, 100.}	inf	144.	100.328	{79.562, 121.259}	30.328	0.162572	newly	106.	98.81	{76., 121.}	13.9717	0.0663738	Reff	0.755633	0.770139	{0.767307, 0.772894}	1.91966	ar	4758.	4560.13	{4435., 4721.}	4.23897
15	R0	{1.31629, 9.97481, 2.}	{1.25271, 3.3473, 0.}	{{1.22544, 3.07157, 0.}, {1.28596, 3.98513, 0.}}	{4.83053, 66.4425, 100.}	inf	282.	94.4678	{57.8173, 127.732}	66.5008	0.501275	newly	192.	169.14	{111., 229.}	22.6667	0.121124	Reff	0.872437	0.853764	{0.835575, 0.873866}	2.24655	ar	4543.	3620.98	{3405., 3854.}	20.2954
16	R0	{1.36735, 5.74166, 0.1}	{1.34108, 4.20532, 1.92}	{{1.30094, 3.11158, 1.}, {1.3618, 4.98002, 3.}}	{1.92097, 26.7577, 1824.}	inf	112.	74.6405	{51.5652, 98.0514}	33.7115	0.193934	newly	104.	103.39	{70., 144.}	21.125	0.0958346	Reff	0.782534	0.7725	{0.751073, 0.786476}	1.54528	ar	4516.	4464.89	{4345., 4596.}	1.98295
17	R0	{1.27524, 13.2396, 0.1}	{1.08059, 2.87603, 0.93}	{{1.06256, 2.19254, 0.}, {1.11523, 3.74835, 2.}}	{15.2641, 78.277, 876.}	inf	176.	41.7986	{7.50488, 74.654}	76.2508	0.747287	newly	112.	93.61	{17., 168.}	43.3661	0.309883	Reff	1.09887	0.94563	{0.930487, 0.972814}	13.9455	ar	3731.	1671.79	{1237., 2169.}	55.1919
18	R0	{1.21884, 7.71545, 1.}	{1.21312, 3.51866, 0.89}	{{1.21159, 3.07157, 0.}, {1.22544, 3.57392, 1.}}	{0.588636, 54.3947, 11.}	inf	93.	47.174	{28.2676, 67.613}	49.2753	0.335511	newly	79.	82.02	{50., 118.}	30.6835	0.151001	Reff	0.870374	0.871847	{0.868275, 0.877699}	0.312914	ar	3080.	3090.91	{2899., 3319.}	4.28734
19	R0	{1.34879, 6.71464, 0.1}	{1.35297, 3.06018, 2.67}	{{1.30094, 2.5364, 0.}, {1.37363, 3.22945, 3.}}	{1.73465, 54.4252, 2590.}	inf	64.	23.9942	{7.16615, 39.3858}	62.5091	0.518336	newly	49.	42.21	{10., 71.}	39.3673	0.250271	Reff	0.757615	0.765383	{0.736372, 0.780091}	2.19158	ar	4585.	4415.26	{4334., 4497.}	3.70251
20	R0	{1.37284, 5.75036, 0.1}	{1.39051, 1.94786, 0.12}	{{1.39162, 1.89883, 0.}, {1.39162, 1.89883, 0.}}	{1.34144, 66.1263, 212.}	inf	131.	44.8937	{12.7825, 71.7527}	65.73	0.536134	newly	141.	112.78	{32., 181.}	35.9716	0.231726	Reff	0.7784	0.80039	{0.796268, 0.805012}	2.82494	ar	4750.	4412.52	{4260., 4559.}	7.10484
21	R0	{1.29938, 5.36419, 0.1}	{1.28955, 4.02499, 0.32}	{{1.28596, 3.98513, 0.}, {1.29927, 4.21286, 2.}}	{0.979701, 24.9655, 388.}	inf	52.	31.9587	{16.8027, 48.2788}	39.2861	0.250793	newly	62.	44.55	{23., 65.}	32.0484	0.192735	Reff	0.755719	0.752583	{0.748622, 0.758346}	0.73082	ar	4337.	4264.97	{4177., 4348.}	1.99746
22	R0	{1.31882, 7.47626, 0.1}	{1.23827, 3.19506, 0.08}	{{1.22544, 3.07157, 0.}, {1.25474, 3.26445, 0.}}	{6.10779, 57.264, 164.}	inf	105.	36.0634	{11.8752, 61.16}	65.6539	0.543959	newly	86.	65.72	{24., 109.}	38.2326	0.244922	Reff	0.86897	0.824428	{0.81235, 0.836846}	5.1259	ar	3977.	3510.63	{3360., 3661.}	11.7267
23	R0	{1.26215, 3.46375, 0.1}	{1.24721, 2.58886, 0.}	{{1.24751, 2.5364, 0.}, {1.24751, 2.5364, 0.}}	{1.18353, 25.2584, 100.}	inf	13.	15.3584	{4.46345, 26.2717}	57.851	0.25551	newly	14.	31.26	{7., 56.}	143.	0.376041	Reff	0.807776	0.797922	{0.796591, 0.799644}	1.2199	ar	3607.	3657.6	{3597., 3739.}	1.51871
24	R0	{1.16712, 4.19464, 1.}	{1.23357, 2.41132, 0.75}	{{1.19264, 2.01465, 0.}, {1.24751, 2.5364, 3.}}	{5.69383, 42.5143, 125.}	inf	40.	25.4744	{4.96822, 49.1357}	48.3935	0.367862	newly	56.	62.67	{11., 121.}	61.9464	0.317874	Reff	0.834491	0.883793	{0.854979, 0.896025}	5.90801	ar	3000.	3019.99	{2831., 3254.}	4.65967
25	R0	{1.22751, 3.34517, 0.1}	{1.23654, 2.43205, 0.6}	{{1.19264, 2.01465, 0.}, {1.24751, 2.5364, 3.}}	{1.8718, 27.2966, 660.}	inf	37.	38.895	{13.7443, 68.3937}	46.9073	0.224453	newly	58.	89.44	{28., 152.}	78.4138	0.263472	Reff	0.861344	0.867444	{0.837019, 0.878199}	1.85443	ar	3086.	3207.91	{2991., 3446.}	5.24271
26	R0	{1.18747, 5.88083, 1.}	{1.24728, 2.59614, 0.}	{{1.24751, 2.5364, 0.}, {1.24751, 2.5364, 0.}}	{5.03712, 55.8542, 100.}	inf	33.	21.8674	{7.97359, 35.6211}	39.813	0.262143	newly	33.	47.12	{19., 80.}	64.3636	0.22729	Reff	0.772805	0.813121	{0.811135, 0.81525}	5.21676	ar	3614.	3571.93	{3490., 3682.}	2.19286
27	R0	{1.29023, 4.76202, 0.1}	{1.29803, 4.11368, 1.02}	{{1.28596, 3.98513, 0.}, {1.31748, 4.25564, 2.}}	{0.928857, 13.6148, 1018.}	inf	18.	28.2938	{13.4837, 41.1614}	67.0516	0.211892	newly	24.	39.32	{17., 60.}	74.6667	0.232351	Reff	0.74614	0.749312	{0.741556, 0.761375}	0.889395	ar	4245.	4312.07	{4233., 4404.}	1.68881
28	R0	{1.2258, 12.9056, 3.}	{1.21173, 3.56889, 0.99}	{{1.21159, 3.57392, 1.}, {1.21159, 3.57392, 1.}}	{1.14762, 72.3462, 67.}	inf	123.	36.9055	{11.8829, 57.9417}	69.9955	0.597594	newly	72.	63.98	{23., 105.}	36.25	0.216643	Reff	0.891402	0.891604	{0.888766, 0.894534}	0.210659	ar	3639.	2895.7	{2649., 3107.}	20.4259
29	R0	{1.34702, 6.29049, 0.1}	{1.30744, 4.01962, 1.48}	{{1.28596, 3.11158, 0.}, {1.31748, 4.25564, 2.}}	{3.08799, 36.1, 1440.}	inf	82.	47.4283	{26.1891, 66.575}	42.6315	0.27775	newly	86.	66.36	{38., 94.}	28.6512	0.170723	Reff	0.773998	0.755765	{0.741507, 0.764024}	2.57105	ar	4560.	4358.28	{4250., 4476.}	4.49561
30	R0	{1.37931, 6.78741, 1.}	{1.32599, 4.23732, 2.02}	{{1.31748, 4.21286, 1.}, {1.36035, 4.45823, 2.}}	{3.86587, 37.5709, 102.}	inf	58.	30.7996	{13.3618, 47.4429}	47.1837	0.327421	newly	51.	41.09	{16., 65.}	33.1569	0.203331	Reff	0.764552	0.738599	{0.732155, 0.757386}	3.39448	ar	4667.	4504.9	{4433., 4577.}	3.49004
31	R0	{1.16062, 4.47529, 0.1}	{1.23163, 3.00767, 0.03}	{{1.22544, 2.5364, 0.}, {1.24751, 3.26445, 0.}}	{6.1185, 32.7938, 124.}	inf	38.	38.8973	{15.7247, 58.2796}	34.876	0.177987	newly	49.	78.36	{24., 124.}	76.6939	0.254869	Reff	0.82288	0.873116	{0.866314, 0.887036}	6.105	ar	3037.	3140.68	{2933., 3351.}	4.98452
32	R0	{1.37647, 18.7122, 2.}	{1.24427, 3.14791, 0.}	{{1.22544, 3.07157, 0.}, {1.25474, 3.26445, 0.}}	{9.60424, 83.1772, 100.}	inf	359.	53.4059	{28.6636, 75.0243}	85.1237	0.857218	newly	137.	99.64	{50., 137.}	31.0219	0.185636	Reff	0.886034	0.83896	{0.824561, 0.848377}	5.31288	ar	4824.	3526.17	{3360., 3718.}	26.9036
33	R0	{1.35084, 6.08841, 0.1}	{1.3618, 4.98002, 1.}	{{1.3618, 4.98002, 1.}, {1.3618, 4.98002, 1.}}	{0.811586, 18.205, 900.}	inf	48.	48.5754	{34.1592, 63.1891}	20.0512	0.0893156	newly	49.	56.28	{37., 78.}	27.1429	0.10677	Reff	0.722834	0.728623	{0.726523, 0.730538}	0.800746	ar	4759.	4775.37	{4699., 4864.}	1.06976
34	R0	{1.36912, 6.316, 1.}	{1.24751, 2.5364, 0.}	{{1.24751, 2.5364, 0.}, {1.24751, 2.5364, 0.}}	{8.88213, 59.8416, 100.}	inf	145.	45.5641	{13.0657, 77.0833}	68.5765	0.585219	newly	140.	90.58	{23., 146.}	40.9143	0.298858	Reff	0.820103	0.759665	{0.755725, 0.763716}	7.36954	ar	4537.	4045.98	{3903., 4189.}	10.8226
35	R0	{1.35831, 7.09038, 1.}	{1.35849, 5.22152, 1.19}	{{1.34436, 4.98002, 1.}, {1.3618, 6.25107, 2.}}	{0.403469, 26.3577, 19.}	inf	73.	56.5169	{37.6573, 79.9788}	27.2083	0.149316	newly	59.	63.33	{44., 83.}	22.8644	0.0998647	Reff	0.752504	0.754848	{0.743836, 0.759248}	0.719343	ar	4667.	4604.22	{4512., 4701.}	1.86887
36	R0	{1.28315, 5.89938, 0.1}	{1.34698, 3.36076, 2.8}	{{1.30094, 3.11158, 2.}, {1.36035, 4.25564, 3.}}	{4.97429, 43.032, 2702.}	inf	40.	23.0831	{5.69633, 40.1229}	44.6492	0.343612	newly	43.	37.53	{11., 71.}	45.5581	0.270069	Reff	0.715356	0.75322	{0.729482, 0.763575}	5.29294	ar	4460.	4473.01	{4400., 4554.}	1.18946
37	R0	{1.19303, 2.75113, 0.1}	{1.2072, 2.05363, 2.51}	{{1.19264, 2.01465, 0.}, {1.24751, 2.5364, 3.}}	{1.35577, 25.3532, 2444.}	inf	5.	15.344	{3.56469, 29.7827}	217.071	0.441022	newly	9.	41.45	{8., 83.}	369.889	0.589574	Reff	0.839297	0.848016	{0.836229, 0.877574}	1.42582	ar	3142.	3061.02	{2970., 3179.}	3.57161
38	R0	{1.36033, 7.8088, 1.}	{1.333, 4.35898, 1.86}	{{1.30222, 3.22945, 1.}, {1.3618, 4.98002, 3.}}	{2.16732, 44.1786, 98.}	inf	165.	66.1879	{35.9228, 86.8927}	59.8861	0.423766	newly	127.	88.56	{52., 120.}	31.7008	0.186823	Reff	0.778789	0.776299	{0.763168, 0.793179}	1.7108	ar	4941.	4385.22	{4252., 4512.}	11.2483
39	R0	{1.27353, 3.70845, 0.1}	{1.24758, 2.59807, 0.}	{{1.24751, 2.5364, 0.}, {1.24751, 2.5364, 0.}}	{2.03786, 29.9419, 100.}	inf	7.	9.47234	{2.90182, 16.7979}	67.3604	0.253425	newly	14.	20.43	{5., 39.}	82.7857	0.307104	Reff	0.800796	0.784168	{0.783163, 0.78504}	2.07638	ar	3725.	3751.6	{3712., 3798.}	0.884295
40	R0	{1.19983, 12.1486, 0.1}	{1.17082, 5.77111, 1.}	{{1.17082, 5.77111, 1.}, {1.17082, 5.77111, 1.}}	{2.41747, 52.4957, 900.}	inf	140.	46.0995	{32.0953, 59.7148}	67.0718	0.495551	newly	71.	52.11	{36., 68.}	28.2394	0.155971	Reff	0.902632	0.891805	{0.89017, 0.89342}	1.19946	ar	3619.	2682.36	{2468., 2957.}	25.8812
41	R0	{1.20935, 2.67169, 0.1}	{1.26215, 1.63584, 2.28}	{{1.24751, 1.35135, 0.}, {1.2663, 2.5364, 3.}}	{4.36571, 38.7712, 2228.}	inf	0.1	6.04351	{1.80103, 10.9206}	5943.51	1.6977	newly	0.1	19.55	{3., 41.}	19450.	2.13771	Reff	0.794664	0.828593	{0.819068, 0.831794}	4.26966	ar	3429.	3464.97	{3432., 3524.}	1.04899
42	R0	{1.23313, 4.74781, 1.}	{1.28319, 3.93116, 0.03}	{{1.28596, 3.98513, 0.}, {1.28596, 3.98513, 0.}}	{4.07178, 17.2005, 101.}	inf	65.	44.8042	{25.2451, 66.0486}	33.0078	0.208031	newly	90.	67.2	{33., 103.}	31.4	0.189705	Reff	0.791546	0.82627	{0.824164, 0.83037}	4.38909	ar	3835.	3750.52	{3610., 3911.}	3.11864
43	R0	{1.26105, 2.39713, 0.1}	{1.25965, 1.91692, 1.65}	{{1.24751, 1.35135, 0.}, {1.2663, 2.5364, 3.}}	{0.87676, 25.3782, 1642.}	inf	1.	6.77056	{1.74926, 13.7881}	577.056	0.722137	newly	1.	20.04	{3., 50.}	1904.	1.10493	Reff	0.791561	0.789956	{0.782165, 0.79472}	0.851513	ar	3723.	3759.17	{3725., 3821.}	0.971528
44	R0	{1.36948, 8.88006, 0.1}	{1.28159, 3.88424, 0.}	{{1.25474, 3.26445, 0.}, {1.28596, 3.98513, 0.}}	{6.4179, 56.2589, 100.}	inf	166.	72.7614	{48.9779, 96.9525}	56.1679	0.37357	newly	127.	109.	{69., 142.}	21.7953	0.11248	Reff	0.844011	0.801789	{0.786857, 0.807415}	5.00246	ar	4437.	4002.7	{3854., 4151.}	9.87694
45	R0	{1.34882, 3.3791, 0.1}	{1.29249, 2.84735, 1.83}	{{1.24751, 2.5364, 0.}, {1.36035, 3.11158, 3.}}	{4.68749, 15.7364, 1810.}	inf	6.	11.5185	{3.47221, 21.0919}	109.835	0.307482	newly	9.	21.75	{6., 45.}	159.444	0.379449	Reff	0.755069	0.722819	{0.697206, 0.761074}	4.74032	ar	4422.	4439.12	{4402., 4497.}	0.646314
46	R0	{1.23774, 4.34917, 0.1}	{1.25206, 3.20453, 0.}	{{1.22544, 2.5364, 0.}, {1.25474, 3.26445, 0.}}	{1.39561, 26.3185, 100.}	inf	30.	24.8279	{6.50792, 44.9851}	44.3545	0.270686	newly	38.	43.4	{12., 78.}	58.4737	0.273379	Reff	0.787698	0.797453	{0.782468, 0.801483}	1.43031	ar	3685.	3717.69	{3627., 3826.}	1.79783
47	R0	{1.34327, 4.78811, 0.1}	{1.29598, 4.0493, 0.67}	{{1.28596, 3.98513, 0.}, {1.31748, 4.25564, 2.}}	{3.54576, 15.4302, 704.}	inf	76.	62.975	{39.9646, 84.0024}	22.7628	0.118744	newly	94.	88.32	{56., 117.}	21.5319	0.103521	Reff	0.773992	0.74843	{0.73994, 0.761587}	3.33819	ar	4517.	4404.81	{4289., 4531.}	2.795
48	R0	{1.24366, 4.25814, 0.1}	{1.24779, 2.49107, 0.15}	{{1.24751, 2.5364, 0.}, {1.24751, 2.5364, 0.}}	{0.413957, 41.4987, 240.}	inf	38.	32.9466	{12.7978, 53.0806}	38.7566	0.221596	newly	50.	69.7	{23., 122.}	66.56	0.247028	Reff	0.803653	0.806952	{0.803901, 0.809743}	0.524166	ar	3678.	3655.01	{3536., 3777.}	2.32273
49	R0	{1.36017, 7.5415, 1.}	{1.2314, 3.0946, 0.03}	{{1.22544, 3.07157, 0.}, {1.25474, 3.26445, 0.}}	{9.46721, 58.9657, 97.}	inf	152.	54.2613	{26.1007, 86.2455}	64.3018	0.493181	newly	140.	103.71	{45., 166.}	34.95	0.212426	Reff	0.945454	0.867982	{0.859757, 0.884978}	8.19419	ar	4178.	3242.38	{3038., 3447.}	22.394
50	R0	{1.25333, 6.63578, 1.}	{1.23299, 3.10683, 0.01}	{{1.22544, 3.07157, 0.}, {1.25474, 3.26445, 0.}}	{1.67658, 53.1806, 99.}	inf	49.	29.6954	{12.4516, 44.1916}	41.4092	0.285969	newly	43.	55.47	{22., 86.}	56.4884	0.225233	Reff	0.846499	0.835142	{0.828184, 0.849846}	1.51978	ar	3401.	3366.99	{3242., 3500.}	2.75595
